# Effects of Ethanol Exposure during Distinct Periods of Brain Development on Hippocampal Synaptic Plasticity

**DOI:** 10.3390/brainsci3031076

**Published:** 2013-07-19

**Authors:** Anna R. Patten, Joana Gil-Mohapel, Ryan C. Wortman, Athena Noonan, Patricia S. Brocardo, Brian R. Christie

**Affiliations:** 1 Division of Medical Sciences, Island Medical Program, University of Victoria, Victoria, V8P 5C2, British Columbia, Canada; E-Mails: apatten@uvic.ca (A.R.P.); jgil@uvic.ca (J.G.-M.); worty@uvic.ca (R.C.W.); noonana@uvic.ca (A.N.); brocardo@uvic.ca (P.S.B.); 2 Department of Biology, University of Victoria, Victoria, V8P 5C2, British Columbia, Canada; 3 Brain Research Centre and Program in Neuroscience, University of British Columbia, Vancouver, V6T 1Z4, British Columbia, Canada; 4 Department of Cellular and Physiological Sciences, University of British Columbia, Vancouver, V6T 1Z4, British Columbia, Canada

**Keywords:** dentate gyrus, ethanol, fetal alcohol spectrum disorders, fetal alcohol syndrome, hippocampus, long-term potentiation, synaptic plasticity, vulnerability period

## Abstract

Fetal alcohol spectrum disorders occur when a mother drinks during pregnancy and can greatly influence synaptic plasticity and cognition in the offspring. In this study we determined whether there are periods during brain development that are more susceptible to the effects of ethanol exposure on hippocampal synaptic plasticity. In particular, we evaluated how the ability to elicit long-term potentiation (LTP) in the hippocampal dentate gyrus (DG) was affected in young adult rats that were exposed to ethanol during either the 1st, 2nd, or 3rd trimester equivalent. As expected, the effects of ethanol on young adult DG LTP were less severe when exposure was limited to a particular trimester equivalent when compared to exposure throughout gestation. In males, ethanol exposure during the 1st, 2nd or 3rd trimester equivalent did not significantly reduce LTP in the DG. In females, ethanol exposure during either the 1st or 2nd trimester equivalents did not impact LTP in early adulthood, but following exposure during the 3rd trimester equivalent alone, LTP was significantly increased in the female DG. These results further exemplify the disparate effects between the ability to elicit LTP in the male and female brain following perinatal ethanol exposure (PNEE).

## 1. Introduction

Fetal Alcohol Spectrum Disorders (FASD) occur when alcohol is consumed during pregnancy and result in abnormal brain development and long lasting deficits in cognitive function [1,2]. Recent reports have estimated the prevalence of FASD in young school children in the USA and some Western European countries to be as high as 2%–5% [3]. It is thought that FASD is the most common cause of mental retardation and birth defects in the United States [1]. The most severe disorder that results from ethanol exposure during pregnancy is Fetal Alcohol Syndrome (FAS). FAS is a disorder characterized by facial dysmorphologies such as midfacial hypoplasia, wide spaced eyes and a smooth philtrum, growth retardation and central nervous system (CNS) dysfunction resulting in cognitive, motor and behavioural problems [1]. Since FAS was first defined in the 1970’s [2,4] it has been realized that the extent of the damage caused by ethanol can vary due to the timing, frequency and volume of ethanol consumed, as well as the genetics and metabolism of the mother, leading to a wide variability in the severity and symptoms. The disorders that result from prenatal ethanol exposure are now grouped under the umbrella term FASD, which encompasses children who show various forms of central nervous system dysfunction.

The brain is not a static organ, but it can change both physically (structural plasticity) and functionally (synaptic plasticity) depending on environment and experience. In particular, it is believed that alterations in hippocampal synaptic plasticity (*i.e.*, changes in the strength of neuronal connections in the hippocampus) may be involved in information storage and hippocampus-dependent learning and memory [5,6]. One form of synaptic plasticity is long-term potentiation (LTP), which corresponds to the strengthening of a synapse and is characterized by an increase in the size of an evoked postsynaptic potential or current in response to the same stimulus [6]. This results in long lasting neuronal changes involving protein expression and activation and an overall increase in synaptic transmission [7]. It is postulated that in the hippocampus LTP and its counterpart long-term depression (LTD), work together to refine and sculpt memories [8].

The hippocampus is one of the major brain regions affected in FASD [9], and both animals [10,11,12,13,14,15,16,17,18] and children [19,20,21,22] that were exposed to ethanol during the period of brain development show learning and memory impairments. Previous studies from our laboratory and others have also indicated that prenatal ethanol exposure can cause long lasting deficits in hippocampal synaptic plasticity [14,15,23,24,25,26,27]. In particular, our laboratory has shown that LTP is decreased in the dentate gyrus (DG) of both adolescent [23] and young adult [24,28] males that were exposed to ethanol throughout gestation. 

It is well known that the sensitivity of the CNS to the effects of ethanol varies throughout the perinatal period, with specific cell types being more sensitive during certain stages of development [29,30,31,32]. For example, previous studies from our laboratory have shown that the effects of perinatal ethanol exposure (PNEE) on oxidative stress in the young adult rat hippocampus are more pronounced when exposure occurred during all three-trimester equivalents [33] as compared to the 1st and 2nd trimester equivalents combined [34]. This indicates that the 3rd trimester equivalent (which corresponds to the brain growth spurt in humans; [31,35]) might be particularly sensitive to the effects of ethanol on oxidative stress. However, exposure during the 1st and 2nd trimester equivalents combined (*i.e.*, throughout gestation in rats) is enough to induce a long-lasting impairment in hippocampal synaptic plasticity that can be detected in early adulthood [24,28]. Furthermore, a recent large-scale clinical study has shown that drinking during the 1st trimester (when many women are not yet aware of their pregnancy) has a strong association with signs of alcohol damage to the fetus [36]. A different study revealed that while pregnant women can eliminate ethanol from their blood faster during the 2nd trimester, this teratogen is cleared from the amniotic fluid at a much slower rate during this period, indicating that the fluid may act as an ethanol reservoir [37]. Therefore, it is of particular relevance to further characterize the effects that ethanol exposure might have during particular periods of brain development. 

The aim of this study was to determine whether ethanol exposure during a specific trimester equivalent renders the DG of the hippocampus more vulnerable to deficits in LTP later on, when animals reach early adulthood. As previous research from our laboratory has demonstrated the existence of sexually dichotic effects of PNEE on LTP [23,24,28], the present study evaluated how ethanol-exposure during the 1st, the 2nd, or the 3rd trimester equivalent affected LTP both in the male and female young adult DG. Identifying intervals during the period of brain development of enhanced vulnerability to the effects of ethanol exposure on synaptic plasticity may further elucidate the mechanisms underlying the wide range of cognitive manifestations that are associated with FASD. 

## 2. Results and Discussion

### 2.1. Effects of Ethanol Exposure on Body Weight and Litter Size

Weight data was taken from pregnant dams on gestational days (GDs) 1, 7, 14 and 21. The percentage weight gain over the course of pregnancy did not differ between perinatal conditions [*F*(6, 21) = 1.7, *p* = 0.17]. However, there was a significant effect of perinatal condition on litter size [*F*(6, 21) = 2.8, *p* = 0.04], with 1st trimester pair-fed animals having larger litters as compared to 1st trimester ethanol exposed animals (*p* < 0.05). These results are summarized in Table 1.

Offspring weight was determined during the lactation period on postnatal days (PNDs) 2, 6, 10, 14, 18 and 22 to determine whether perinatal condition altered offspring weight gain. A repeated measures analysis of variance (ANOVA) revealed that there was no significant effect of sex [*F*(6, 37) = 2.0, *p* = 0.99], and therefore, data from both males and females were combined and a repeated measures ANOVA for perinatal condition (*ad libitum*, pair-fed1, pair-fed2, pair-fed3, ethanol1, ethanol2, ethanol3) revealed a significant effect of perinatal condition [*F*(36, 196) = 3.0, *p* < 0.0001]. Further *post-hoc* analysis revealed that at PND 2, 1st trimester pair-fed animals weighed significantly less than both *ad libitum* (*p <* 0.05) and 1st trimester ethanol-exposed animals (*p* < 0.001). There were no significant differences in weight between PNDs 6 and 14 for all conditions. At PND 18, 3rd trimester pair-fed animals weighed significantly more than both 1st trimester pair-fed animals (*p* < 0.05) and 2nd trimester pair-fed animals (*p* < 0.01). This difference remained at PND 22, with 3rd trimester pair-fed animals still weighing significantly more than 1st trimester pair-fed animals (*p* < 0.001) and 2nd trimester pair-fed animals (*p* < 0.01). These results are summarized in Table 2.

**Table 1:** Effect of perinatal ethanol exposure on dam weight gain during pregnancy and litter size. There were no significant differences in weight gain over pregnancy between perinatal conditions. 1st trimester pair-fed animals had significantly more pups than 1st trimester ethanol exposed animals, but all other groups had comparable litter sizes. Results are expressed as means ± standard error of the mean (SEM). Results are considered statistically significant if *p* < 0.05. * *p* < 0.05, as compared to 1st trimester ethanol exposed dams.

	% weight gain during pregnancy	Number of pups per litter
***Ad libitum***	61.6 ± 11.3	13.7 ± 2.3
**Ethanol 1st**	74.4 ± 3.8	12.8 ± 1.3
**Pair-fed 1st**	66.3 ± 9.3	18 ± 1.7 *
**Ethanol 2nd**	51.1 ± 3.2	15.3 ± 0.5
**Pair-fed 2nd**	63.1 ± 4.3	13.0 ± 0.6
**Ethanol 3rd**	65.4 ± 4.3	14.0 ± 0.8
**Pair-fed 3rd**	69.7 ± 5.2	15.6 ± 0.2

**Table 2:** Effect of perinatal ethanol exposure on offspring body weight. A repeated measures ANOVA revealed that there was no significant effect of sex and therefore data from both males and females were combined. A significant main effect of perinatal condition was observed (see text for statistical details). Results are expressed as means ± SEM. Results are considered statistically significant if *p* < 0.05. * *p* < 0.05 compared to *ad libitum* controls and 1st trimester ethanol exposed animals (postnatal day (PND) 2). ^#^ *p* < 0.05 compared to 3rd trimester pair-fed animals (PND 18). ^$$^ *p* < 0.01 compared to 3rd trimester pair-fed animals (PND 22).

	*Ad libitum*	Ethanol-exposed			Pair-fed		
	Weight (g)	1st	2nd	3rd	1st	2nd	3rd
**PND2**	8.0 ± 0.3	8.3 ± 0.3	7.5 ± 0.2	8.1 ± 0.2	6.9 ± 0.4 *	7.8 ± 0.2	7.7 ± 0.2
**PND5**	15.0 ± 0.5	14.6 ± 0.4	14.5 ± 0.4	14.9 ± 0.4	13.6 ± 0.4	14.4 ± 0.4	14.6 ± 0.4
**PND10**	23.6 ± 0.8	23.3 ± 0.6	23.2 ± 0.7	24.0 ± 0.7	22.2 ± 0.7	23.0 ± 0.7	24.8 ± 0.6
**PND14**	33.3 ± 1.1	32.8 ± 0.9	33.4 ± 0.9	34.2 ± 1.0	32.1 ± 0.9	32.0 ± 0.9	35.8 ± 0.9
**PND18**	43.7 ± 1.4	41.8 ± 1.2	43.2 ± 1.2	45.1 ± 1.5	41.2 ± 1.2 ^#^	41.0 ± 1.2 ^#^	47.2 ± 1.3
**PND22**	60.4 ± 1.9	60.4 ± 1.7	59.8 ± 1.7	63.3 ± 1.9	54.7±1.7 ^$$^	59.1±1.7 ^$$^	67.3 ± 1.7

When animals reached experimental age (PNDs 55–70), a significant main effect of sex was observed [*F*(1, 101) = 1481.6, *p* < 0.0001], with males being significantly heavier than females (*p* < 0.001) and data from males and females was subsequently analyzed separately. Nevertheless, there was no significant main effect of perinatal condition on body weight at experimental age both in males [*F*(6, 50) = 2.05, *p* = 0.08] and females [*F*(6, 51) = 1.07, *p* = 0.39]. These results are summarized in Table 3.

**Table 3:** Effect of perinatal ethanol exposure on offspring body weight at experimental age. When reaching early adulthood, males weighed significantly more than females and therefore their weights were analyzed separately. There were no significant effects of perinatal condition on body weight in either males or females (see text for statistical details). Results are expressed as means ± SEM. Results are considered statistically significant if *p* < 0.05.

	Weight (g)	Male	Female
***Ad libitum***	***--***	413.1 ± 6.2	271.5 ± 14.3
**Ethanol-exposed**	**1st**	417.5 ± 5.2	281.1 ± 11.4
	**2nd**	400.0 ± 7.0	267.0 ± 6.1
	**3rd**	408.4 ± 4.7	257.1 ± 5.4
**Pair-fed**	**1st**	413.0 ± 1.6	263.1 ± 3.0
	**2nd**	413.2 ± 3.4	269.7 ± 4.0
	**3rd**	421.6 ± 4.1	272.1 ± 5.7

### 2.2. Intoxication Levels

Peak blood alcohol concentrations (BACs) were measured from blood taken two hours after the dark cycle commenced on GD 10 or GD 20 for 1st or 2nd trimester equivalent exposure, respectively. Blood taken 2 h following the last feeding of the ethanol diet on PND 10 was used to measure peak BAC levels for 3rd trimester equivalent exposure. The BACs for 1st, 2nd and 3rd trimester equivalent exposed animals were 91.6 mg/dL, 94.3 mg/dL, and 255.1 mg/dL, respectively. These results are in agreement with the literature, with the gavage model (3rd trimester equivalent) producing considerably higher BACs than the prenatal liquid diet exposure model [33,38,39]; for review see [40].

### 2.3. Effects of Ethanol Exposure on Basal Synaptic Transmission and Post-Tetanic Potentiation

To determine whether ethanol exposure during a specific trimester equivalent affected basal synaptic transmission, input/output (I/O) analysis was performed. In all animals, the slope of the field excitatory post-synaptic potential (fEPSP) significantly increased with increasing stimulation [repeated measures ANOVA: *F*(4, 288) = 287.2, *p* < 0.0001]. Perinatal condition had no significant effect on I/O function repeated measures ANOVA: [*F*(6, 72) = 0.51, *p* = 0.80], regardless of sex [*F*(1, 72) = 0.005, *p* = 0.94] (data not shown).

To assess the effects of ethanol exposure during specific trimester equivalents on short-term plasticity, post-tetanic potentiation (PTP; defined as the first minute following stimulation) was assessed. In males, a one-way ANOVA revealed a significant main effect of perinatal condition [*F*(6, 47) = 2.78, *p* = 0.02]. *Post-hoc* analyses showed that PTP was significantly reduced in males exposed to ethanol during the 1st trimester equivalent when compared to males exposed to ethanol during the 3rd trimester equivalent (*p* < 0.05). In females, a one-way ANOVA revealed no significant effects of perinatal condition [*F*(6, 45) = 1.73, *p* = 0.14]. Additionally, the results for each trimester equivalent were analysed individually. In males, there was a significant main effect of perinatal condition on PTP in the 1st trimester equivalent exposure group [*F*(2, 20) = 4.05, *p* = 0.03], but *post-hoc* analysis showed only a trend towards a decrease in the ethanol-exposed animals when compared to the *ad libitum* controls (*p* = 0.06). There was no significant main effects of perinatal condition on PTP in the 2nd [*F*(2, 20) = 1.28, *p* = 0.3] or 3rd [*F*(2, 17) = 1.3, *p* = 0.3] trimester equivalent exposure groups. In females, there was no significant main effect of perinatal condition on PTP in the 1st [*F*(2, 18) = 0.3, *p* = 0.7], 2nd [*F*(2, 18) = 2.0, *p* = 0.17] or 3rd [*F*(2, 19) = 2.4, *p* = 0.12] trimester equivalent exposure groups. 

### 2.4. Effects of Ethanol Exposure on Young Adult DG LTP

We have previously established that PNEE young adult males (PNDs 55–70) show a significant reduction in LTP in the DG, whereas young adult female animals do not show such deficits [24,28]. The present study aimed to determine whether LTP is affected differently if exposure to ethanol occurs only during a specific trimester equivalent in both male and female offspring.

**Figure 1:**
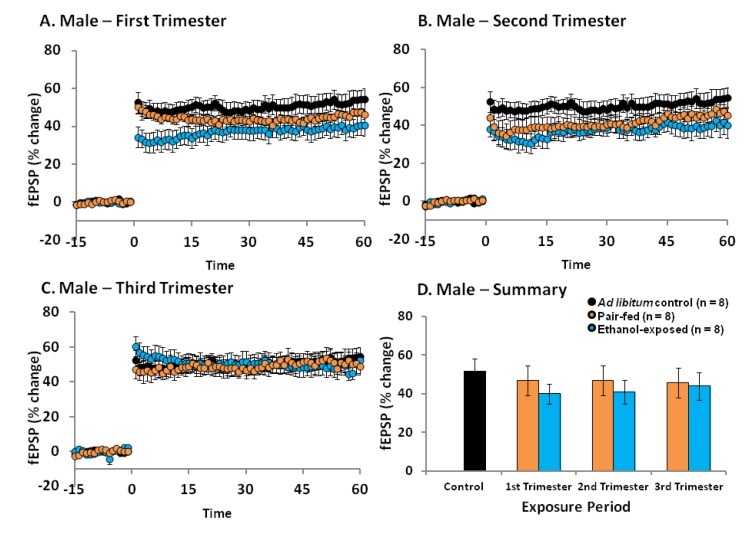
The effects of perinatal ethanol exposure (PNEE) during specific trimester equivalents on long-term potentiation (LTP) in the dentate gyrus (DG) of young adult male rats. PNEE during either the 1st (**A**), 2nd (**B**), or 3rd (**C**) trimester equivalents does not result in a significant reduction in DG LTP. (**D**) Summary of LTP results calculated by assessing the initial phase of the excitatory post-synaptic potentiation (EPSP) slope (10%–80%) at 55–60 min post-theta burst stimulation (TBS). Results are presented as means ± SEM and were considered statistically significant when *p* < 0.05.

In male offspring, a one-way ANOVA for perinatal condition (control, pair-fed1, pair-fed2, pair-fed3, ethanol1, ethanol2, ethanol3) revealed that there was no significant effect of perinatal condition on LTP [*F*(6, 53) = 0.601, *p* = 0.728; Figure 1]. Additionally, when the results for each trimester equivalent were analysed individually, 3 out of 8 males that were exposed to ethanol during the 1st trimester equivalent alone showed less than 30% LTP, whereas 2 out of 8 males that were exposed to ethanol during the 2nd trimester equivalent alone showed less than 30% LTP (data not shown). This is in contrast to *ad libitum* control males, pair-fed males and 3rd trimester equivalent ethanol-exposed males, where no animals showed less than 30% LTP. However, additional statistical analysis of each individual trimester equivalent failed to reveal any significant effects of perinatal condition during either the 1st [*F*(2, 20) = 1.63, *p* = 0.22], 2nd [*F*(2, 20) = 0.96, *p* = 0.40] or 3rd [*F*(2, 19) = 0.12, *p* = 0.89] trimester equivalent exposure groups. These results indicate that overall, ethanol exposure during a single trimester equivalent is not enough to cause reliable long-term deficits in LTP in the male DG.

**Figure 2:**
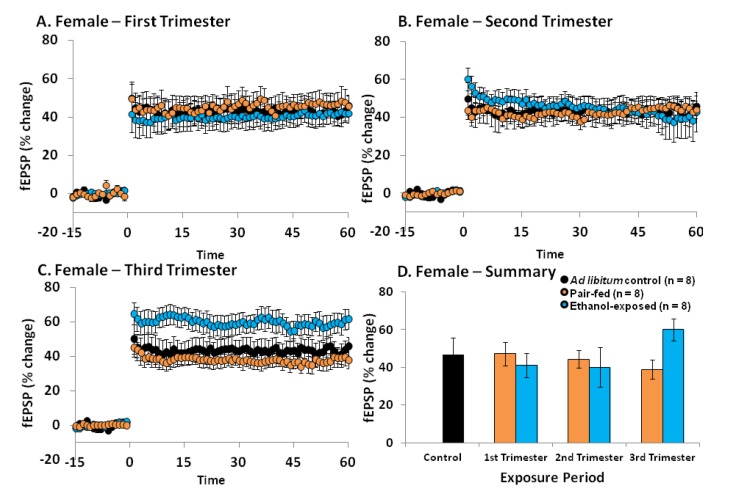
The effects of PNEE during specific trimester equivalents on LTP in the DG of young adult female rats. PNEE during either the 1st (**A**) or 2nd (**B**) trimester equivalents does not result in a significant reduction in DG LTP. (**C**) PNEE during the 3rd trimester equivalent significantly increased LTP in females when compared to their respective *ad libitum* (*p* < 0.01) and pair-fed (*p* < 0.01) controls (see text for additional details on the statistical analysis). (**D**) Summary of LTP results calculated by assessing the initial phase of the EPSP slope (10%–80%) at 55–60 min post-TBS. Results are presented as means ± SEM and were considered statistically significant when *p* < 0.05.

In females, a one-way ANOVA (control, pair-fed1, pair-fed2, pair-fed3, ethanol1, ethanol2 or ethanol3) showed no significant effect of perinatal condition [*F*(6, 45) = 1.45, *p =* 0.22] (Figure 2). Interestingly, if the results for each trimester equivalent were individually analyzed, no significant effects of perinatal condition were obtained for either the 1st [*F*(2, 19) = 0.21, *p* = 0.81] or 2nd [*F*(2, 18) = 0.09, *p* = 0.92] trimester equivalent exposure groups, but a significant effect was revealed for the 3rd trimester equivalent exposure group [*F*(2, 18) = 3.73, *p* < 0.05]. *Post-hoc* analyses further demonstrated that when ethanol exposure occurred during the third trimester equivalent, ethanol exposed females have increased LTP as compared to both *ad libitum* control (*p* < 0.05) and pair-fed (*p* < 0.05) females.

### 2.5. Discussion

Previous research from our laboratory has indicated that ethanol exposure throughout gestation (*i.e.*, 1st and 2nd trimester equivalents in rats) causes long-lasting deficits in DG LTP in young adult males (PNDs 55–70) [23,24,28]. Females on the other hand, show an enhanced capacity for DG LTP in adolescence (PNDs 30–35) [23], a phenomenon which disappears in early adulthood (PNDs 55–70), where LTP levels are comparable to those of control females [24,28].

In this study we examined which periods during brain development are more susceptible to ethanol exposure with regards to the ability to elicit LTP in the DG of the hippocampus. Not surprisingly, we found that in males the long-term effects of ethanol on DG synaptic plasticity were considerably less robust when exposure was limited to an individual trimester equivalent (present study) as compared to continuous exposure throughout gestation [23,24,28]. In fact, for all three exposure periods (*i.e.*, either 1st, 2nd, or 3rd trimester equivalents), there were no statistically significant differences in the ability to elicit LTP among ethanol-exposed, pair-fed, and *ad libitum* control males. In a 1990 study conducted by Tan *et al.* [41], an *in vitro* examination of LTP in the CA1 region of the hippocampus showed that when alcohol exposure (through a liquid diet with either 35% or 17.5% ethanol) occurred between GDs 8 and 22 (*i.e.*, late 1st trimester–2nd trimester exposure) no changes in LTP were observed [41]. These results, in combination with the results from the present study, indicate that alcohol exposure during the second trimester does not reduce LTP in either the DG or CA1 sub-regions of the hippocampus. These results are also in line with previous studies that have shown that ethanol exposure during the 3rd trimester equivalent does not affect synaptic plasticity in the CA1 [42,43] and that ethanol exposure only during the 1st, 2nd, or 3rd trimester equivalent does not result in deficits in spatial navigation [42,44]. Overall, these results indicate that ethanol exposure during a limited period of brain development is not enough to robustly alter hippocampal synaptic plasticity (and possibly hippocampal-sensitive behaviours; [42,44]) when animals reach early adulthood. 

Of note, a degree of variability was observed in the animals that were exposed to ethanol during either the 1st or 2nd trimester equivalents, with 2–3 out of 8 males that were exposed to ethanol during either the 1st or 2nd trimester equivalent alone showing less than 30% LTP. In contrast, no such variability was noted among the males that were exposed to ethanol during the 3rd trimester equivalent alone. The reasons underlying the variability that was obtained following exposure during the 1st or 2nd trimester equivalents are likely related with the different modes of ethanol administration that were used for the 1st and 2nd trimester equivalent groups (*i.e.*, voluntary consumption of a liquid diet containing ethanol by the pregnant dam) and the 3rd trimester equivalent group (*i.e.*, intragastric intubation of the offspring with a pre-determined amount of ethanol). 

Indeed, due to the fact that in rats the third trimester equivalent of brain development occurs postnatally (from PNDs 1 to 10), a liquid diet cannot be administered across all three trimester equivalents. While it is possible to administer the liquid diet to lactating mothers during the third trimester equivalent, it is uncertain how much ethanol crosses into the breast milk, and whether the pups will suckle if ethanol is present in the milk (for review see [40]). Previous studies have used either the vapour inhalation method or the gavage (*i.e.*, gastric intubation) method to expose rat pups during the third trimester equivalent and while both methods are associated with high BACs, the gavage method better models the route of ethanol consumption in humans (*i.e.*, oral administration), and is therefore preferred. Additionally, the gavage model has the added advantage of controlling for the timing of exposure and dosing of ethanol, thus significantly reducing variability within groups (for review see [40]). On the other hand, while the liquid diet model usually produces BACs that more closely reflect those obtained with moderate drinking (*i.e.*, more representative of the vast majority of the human population that consumes alcohol during pregnancy [45]), it is also associated with an increased variability when compared with the gavage model. For example, the time of exposure, the daily period of exposure, or the time during the day when the highest BAC is reached may differ dramatically among pregnant dams that have free access to an ethanol liquid diet and these factors may differentially affect their offspring (for review see [40]). Thus, it is not surprising that higher group variability was observed in the 1st and 2nd trimester equivalent groups. Future studies utilizing the gavage model for all periods of exposure may provide a better understanding of how LTP is affected during each trimester equivalent alone.

Interestingly, exposure to a high BAC (255.1 mg/dL) during the 3rd trimester equivalent also failed to induce significant changes in LTP in the DG of young adult male rats. This may be related to the fact that in this case, exposure to ethanol occurred after the population of DG precursor cells (*i.e.*, the radial glia cells) had been generated. Indeed, this population of precursor cells is formed during the 2nd trimester equivalent [46,47], and therefore if animals are only exposed to ethanol during the 3rd trimester equivalent, this process is not affected. On the other hand, while increased cell proliferation is known to occur in the DG during the 3rd trimester equivalent (*i.e.*, the period of brain growth spurt) [35] and exposure to ethanol during this period may disrupt this process by increasing apoptosis of the newly generated cells, previous studies from our laboratory indicated that ethanol exposure during the three trimester equivalents does not impact the rate of cell proliferation and in fact increases the rate of neuronal differentiation in the adult DG [38,39]. Thus, despite a potential ethanol-induced increase in apoptosis during the third trimester, it is possible that the size of the DG granule cell population can be restored due to a compensatory mechanism of ethanol-induced increased neuronal differentiation [38,39]. This might explain why, by the time LTP was examined (*i.e.*, when animals reached adulthood), no significant differences in LTP were detected between control and ethanol-exposed animals. On the other hand, when exposure occurs during gestation (and concomitant with the generation of DG precursor cells), even though lower BACs are achieved, the DG population of precursor cells may be intrinsically affected by ethanol and not be able recover. In this case, even though the rate of adult hippocampal neurogenesis is not affected by ethanol exposure [38,39], the precursor cells responsible for producing the new granule cells may be intrinsically damaged and hence all daughter cells will also be potentially affected and unable to function properly. This may then explain why significant deficits in DG LTP are observed when ethanol exposure via an ethanol-containing liquid diet occurs throughout gestation (*i.e.*, 1st and 2nd trimester equivalents combined) [24,28].

As well as LTP, PTP was also examined in these male offspring. PTP describes an enhancement of transmitter release lasting for minutes after HFS-induced LTP of synapses in the MPP of the DG. This enhancement is largely due to an increase in intracellular Ca^2+^ concentration in the pre-synaptic terminal during the stimulus trains. The increased residual Ca^2+^ combines with the Ca^2+^ influx that is triggered by the following action potential to enhance neurotransmitter release [48]. Our findings indicate that males exposed to ethanol during the 1st trimester equivalent have a decreased capacity for PTP compared to *ad libitum* control males which may indicate differences in neurotransmitter release probabilities. However, this difference does not cause significant changes in the ability to elicit LTP (Figure 1). These presynaptic differences and their potential effects on neurotransmission and behaviour should be explored in future studies.

Previous studies have shown that PNEE females show increased LTP during adolescence (PNDs 30–35) [23] and no differences in LTP during early adulthood (PNDs 55–70) [24,28]. It is therefore not surprising that PNEE during either the 1st or the 2nd trimester equivalent alone did not impact LTP in the young adult female DG (Figure 2). However, when PNEE occurred during the 3rd trimester equivalent alone, a significant increase in DG LTP was detected when compared to *ad libitum* control and pair-fed females (Figure 2). This increase is similar to that observed in the DG of adolescent females that were exposed to ethanol throughout gestation (*i.e.*, 1st and 2nd trimester equivalents combined) [23]. Reasons for this increase are currently unknown but may be related to a dysregulation of estrogen levels with PNEE. Indeed, PNEE females exhibit an increased hypothalamic-pituitary-adrenal (HPA) sensitivity to estrogen, and estrogen levels are higher during proestrous in PNEE females compared to controls [49]. Furthermore, a recent study suggests that PNEE may lead to increased basal estrogen levels [50] and high levels of this hormone are known to increase LTP [51,52], possibly explaining the increase in DG LTP observed in PNEE females. Why this enhancement is only observed after ethanol exposure during the 3rd trimester is currently unknown, but may be related to the fact that estrogen does not begin to be produced in the ovaries until PND5 (*i.e.*, during the 3rd trimester equivalent) [53]. Perhaps the most striking result is that by examining ethanol exposure during distinct periods of brain development we have uncovered a defined time window during which exposure to ethanol results in enhanced LTP in the young adult female DG.

Of note, while enhanced LTP is often associated with increased learning and memory, this is not always the case. In fact, impairments in spatial performance have been accompanied by increased LTP in the CA1 [54], and Jeffery *et al.* (1995) found a negative correlation between magnitude of LTP in the DG and spatial memory performance [55]. Therefore, no extrapolations should be made with regards to the possible effects of ethanol exposure during the 3rd trimester equivalent and learning and memory abilities in females. In fact, learning and memory deficits are commonly observed in females following PNEE, particularly with 3rd trimester exposure [11,12,17]. 

## 3. Experimental Section

### 3.1. Animals and Breeding

All animal procedures were performed in accordance with the University of Victoria and the Canadian Council for Animal Care policies. 

**Figure 3:**
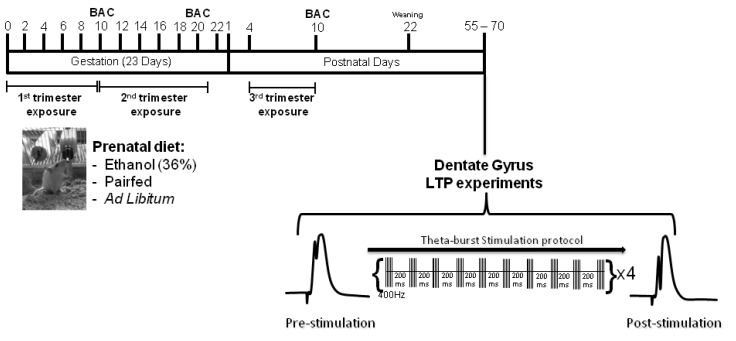
Experimental Timeline. On GD1 pregnant dams were assigned to one of seven treatment groups: *Ad libitum* controls, 1st trimester equivalent ethanol exposure, 1st trimester equivalent pair-fed, 2nd trimester equivalent ethanol exposure, 2nd trimester equivalent pair-fed, 3rd trimester equivalent ethanol exposure, or 3rd trimester equivalent pair-fed (see text for a detailed explanation of the various groups). Blood samples to assess BAC were taken on GD 10 for the 1st trimester equivalent exposure condition, GD20 for the 2nd trimester equivalent exposure condition, and PND 10 for the 3rd trimester equivalent exposure condition. When animals reached experimental age (PNDs 55–70) they were used for *in vivo* electrophysiological experiments to examine LTP in the DG of the hippocampus. Basal recordings were first obtained by administering a pulse (0.12 ms in duration) at 0.067 Hz (pre-stimulation). Once a stable baseline was observed for at least 15 min, LTP was induced by applying TBS consisting of 10 bursts of 5 pulses at 400 Hz with an inter-burst interval of 200 ms, which was repeated 4 times at 30 s intervals. The pulse duration was changed to 0.25 ms during TBS. Following TBS, baseline stimulation resumed for 60 min (post-stimulation).

Abbreviations: Blood alcohol concentration (BAC); Dentate gyrus (DG); Gestational day (GD); Long-term potentiation (LTP); Postnatal day (PND); Theta burst stimulation (TBS).

Four Male (300–350 g) and 30 virgin female (250–275 g) Sprague-Dawley rats were obtained from Charles River Laboratories (Quebec, Canada). Females were housed in pairs and breeding males were housed individually in clear polycarbonate cages (46 × 24 × 20 cm) with Carefresh contact bedding (Absorption Corp., Bellingham, WA, USA). The room was maintained on a 12-h light:dark cycle (lights on at 7 a.m.) with constant humidity and temperature (22 °C). Following an acclimation period in the unit for at least one week, females and males were housed together and a vaginal smear using 0.9% sodium chloride (NaCl) was performed at the beginning of each light cycle to determine pregnancy. An Olympus Microscope with a 10× objective (Olympus CX21, Center Valley, PA, USA) was used to detect the presence of sperm, which indicated GD 1. The female was immediately removed to a private container supplied with nesting material and placed in one of seven prenatal treatment groups (Figure 3). 

### 3.2. Prenatal Diets

On GD1 pregnant dams were randomly assigned to one of seven groups (4 dams per treatment group).

**1st or 2nd trimester equivalent ethanol exposure**—*Ad libitum* access to a liquid diet containing 35.5% ethanol derived calories during either the 1st trimester equivalent (GDs 1–10) or the 2nd trimester equivalent (GDs 11–21) of pregnancy. Ethanol dams were gradually introduced to the liquid diet over a three-day period (GDs 1–3 or GDs 11–13). On GD1 or 11, one third of the ethanol diet was combined with two thirds of the pair-fed diet (see below), on GD2 or 12, two thirds of the ethanol diet was combined with one third of the pair-fed diet and on GD 3 or 13, 100% of the ethanol diet was supplied to the dam. Dams exposed to ethanol liquid diet during the 1st trimester equivalent received regular chow (Lab Diets 5001, LabDiets, Richmond, IN, USA) from GD 11 onwards. Dams exposed to ethanol liquid diet during the 2nd trimester consumed regular chow from GDs 1 to 10 and then were switched back to a regular chow diet on the final day of pregnancy (GD 22).

**1st or 2nd trimester equivalent pair-fed diet**—The pair-fed groups received a liquid diet with maltose-dextrin isocalorically substituted for the ethanol-derived calories. This liquid diet was not provided *ad libitum*. To control for stress and malnutrition of the ethanol-exposed animals, pair-fed groups received the same amount of food in g/kg/day as their matched ethanol-exposed dams. As above, dams in the 1st trimester equivalent exposure group were given the pair-fed liquid diet between GDs 1 and 10. On GD 11, animals were placed back on a regular chow diet for the remainder of the pregnancy. Dams in the 2nd trimester equivalent exposure group received regular rat chow from GDs 1 to 10 of gestation and were switched to the pair-fed liquid diet between GDs 11 and 21. On GD 22 the dams were switched back to the chow diet.

All liquid diets were given to the animals two hours prior to the beginning of the dark phase each day of the pregnancy. This was done to ensure there were no shifts in the circadian rhythm [56]. When liquid diet bottles were replaced, the bottle from the previous day was weighed to determine the amount of liquid consumed each day. On average animals consume approximately 90–100 g of diet/day. Females were weighed on GDs 1, 7, 14 and 21. 

Liquid diets were obtained from Dyets (Bethlehem, PA, USA) where they are sold as Weinberg/Keiver high protein liquid diet-control (no. 710109) for the pair-fed diet and Weinberg/Keiver high protein liquid diet-experimental (no. 710324) for the ethanol diet. These liquid diets have been nutritionally fortified to ensure that adequate nutrition is provided to the pregnant rats. The ethanol diet contains 1.0 kcal/mL, of which 16.4% are fat derived, 23% are derived from carbohydrate, 25.1% are derived from protein, and 35.5% are ethanol derived. The pair-fed diet is isocaloric, where the carbohydrate-derived calories were increased to 58.5% to substitute for those derived from ethanol [57].

**3rd trimester equivalent ethanol exposure**—Pregnant females were left undisturbed throughout gestation. The day pups were born was considered PND 1. Pups received a dose of 4 g/kg/day of 12% (v/v) ethanol in milk solution between PNDs 4 and 10 (3rd trimester equivalent). Ethanol was dissolved in a nutritional milk solution similar in composition to rat milk [58] and supplemented with a specially formulated vitamin mix (Bio-Serv; Frenchtown, NJ, USA). Solutions were administered by intragastric intubation as previously described by our laboratory [33,37,38,39]. The solution was administered in two separate intubations 2 h apart. An additional feeding of pure milk solution was supplied to ethanol-exposed pups in the evening to counteract the inadequate nutrition, low birth weight and high mortality rate that can be observed with this model of postnatal ethanol exposure [33,38,39].

**3rd trimester equivalent pair-fed diet**—As above, pregnant females were left undisturbed throughout gestation. Between PNDs 4 and 10 (3rd trimester equivalent), pups received a dose of an iso-caloric and iso-volumic maltose-dextrin milk solution. Maltose-dextrin was dissolved in the same nutritional milk solution used to prepare the 3rd trimester equivalent ethanol diet (see above). The solution was administered in two separate intubations 2 h apart. The pair-fed pups were sham-intubated during the third feeding, as extra feeding could cause an excess weight gain in these animals (reviewed in [40]).

***Ad libitum*control**—Pregnant dams had *ad libitum* access to a regular chow diet (Lab Diets 5001, LabDiets, Richmond, IN, USA) throughout pregnancy. Pups were left undisturbed during the 3rd-trimester equivalent.

### 3.3. Blood Alcohol Concentration Assay

For all ethanol-fed dams a single tail blood sample was obtained on GD10 for 1st trimester equivalent exposure or GD20 for 2nd trimester equivalent exposure, approximately two hours after the beginning of the dark phase. For animals that were exposed to ethanol in the 3rd trimester, a tail blood sample was obtained from the pups on PND10, approximately two hours after the last feeding of ethanol-containing diet of the day. Blood was collected in a microcentrifuge tube and allowed to clot overnight at 4 °C. Samples were centrifuged the following day at 3000 *g* for 10 min and the serum (supernatant) was then stored at −20 °C until analysed. Analysis of BACs was performed using the Analox Alcohol Analyzer (Model AMI; Analox Instruments, Lunenberg, MA) and expressed as mg/dL of serum. 

### 3.4. Litters and Weaning

Dams and pups were not disturbed during the initial 24–36 h post-partum to facilitate bonding. Litters were culled to ten pups on PND 2 and all animals were weighed on PNDs 2, 5, 10, 14, 18 and 22. 

Pups were weaned on PND22 and housed in pairs (based on sex) in standard caging and left undisturbed until experimental age (PNDs 55–70) was reached (Figure 3). 

### 3.5. *In Vivo* Electrophysiology

Male and female offspring were used for *in vivo* electrophysiology experiments of DG LTP between the ages of PNDs 55 and 70. To reduce any possible litter effects only two males and two females per litter were included in each experimental group. Eight animals per trimester equivalent, perinatal condition, and sex were used for these experiments. Female subjects were examined each day for at least five days before experimentation using the lavage technique where a vaginal smear using 0.9% NaCl was performed at the beginning of each light cycle to determine the estrous cycle. Females were not used for experimentation if they were in proestrous, where estrogen levels are the highest, as high levels of this hormone may enhance LTP and confound results [51,52]. 

Animals were anaesthetized with urethane (1.5 mg/kg, delivered intraperitoneally, i.p.) and placed on a Kopf stereotaxic apparatus. Body temperature was maintained at 37 ± 0.5 °C throughout the experiment with a grounded homeothermic temperature control unit (Harvard Instruments, MA, USA). Extracellular field potentials were recorded by inserting a 125 μm stainless-steel recording electrode into the hilus of the DG (3.5 mm anterior, 2.0 mm lateral to bregma; [59]) and a 125 μm monopolar stimulating electrode into the ipsilateral medial perforant path (7.4 mm anterior, 4.2 mm lateral to bregma; [59]). Stimulating and recording electrodes were lowered to elicit a maximal response and the stimulation required to induce a 1–2 mV population spike was determined. Prior to basal recordings input/output (I/O) function was assessed by stimulating the tissue with increasing pulse widths (0.04, 0.08, 0.12, 0.16, 0.20 and 0.24 ms in duration) at 0.067 Hz, repeated 5 times at each pulse width. Basal recordings were then obtained by administering a pulse (0.12 ms in duration) at 0.067 Hz. Once a stable baseline was observed for at least 15 min, LTP was induced by applying TBS consisting of 10 bursts of 5 pulses at 400 Hz with an inter-burst interval of 200 ms that was repeated 4 times at 30-s intervals. The pulse duration was changed to 0.25 ms during TBS. Following TBS, baseline stimulation resumed for 60 min (Figure 3), as previously described by us [23,24,28]. Animals were then sacrificed by rapid decapitation.

Signals from the DG were collected on custom-made software (Lee Campbell; Getting Instruments). Signals were amplified (Getting Instruments), filtered (1–3 Hz) and digitized at 5 kHz. For analysis the slope of the rising phase of the fEPSP was used to determine alterations in the level of synaptic efficacy. All fEPSP slope data are presented as the mean percent change from the pre-conditioning baseline ± SEM.

### 3.6. Statistical Analyses

Statistical analysis was performed using the Statistica 7.1 analytical software (StatSoft Inc., Tulsa, OK, USA). All data are presented as means ± SEM. A one-way ANOVA was used to determine the effect of perinatal condition (Ethanol-1, Ethanol-2, Ethanol-3, Pair-fed-1, Pair-fed-2, Pair-fed-3 or *Ad* *libitum* control) on weight gain across pregnancy and litter size. A repeated measures ANOVA for perinatal condition (Ethanol-1, Ethanol-2, Ethanol-3, Pair-fed-1, Pair-fed-2, Pair-fed-3 or *Ad libitum* control) was used to analyze pup weights taken on PNDs 2, 6, 10, 14, 18 and 22. A two-way ANOVA for sex (male or female) and perinatal condition (Ethanol-1, Ethanol-2, Ethanol-3, Pair-fed-1, Pair-fed-2, Pair-fed-3 or *Ad libitum* control) was used to analyze weights at experimental age (PNDs 55–70). A significant main effect of sex was obtained at this age [*F*(1, 101) = 1481.6, *p* < 0.0001], with males weighing significantly more than females (*p* < 0.001). Therefore, male and female data were subsequently analysed separately using one-way ANOVAs. 

A repeated measures ANOVA for perinatal condition (Ethanol-1, Ethanol-2, Ethanol-3, Pair-fed-1, Pair-fed-2, Pair-fed-3 or *Ad libitum* control) was used to analyze I/O data. LTP data were analyzed by assessing the initial phase of the EPSP slope (10%–80%) at 55–60 min post-TBS. Male and female LTP data were analysed separately as previous studies have revealed significant differences between PNEE males and females with regards to DG LTP [24,28]. One-way ANOVAs were used to determine the effect of perinatal condition (Ethanol-1, Ethanol-2, Ethanol-3, Pair-fed-1, Pair-fed-2, Pair-fed-3 or *Ad libitum* control) on PTP and LTP in both males and females. Additionally, PTP and LTP data from each trimester equivalent was individually analyzed with one-way ANOVAs for perinatal condition (Ethanol, Pair-fed, and *Ad libitum* control). *Post-hoc* analyses were conducted using Tukey’s test. A *p* value < 0.05 was considered to be statistically significant.

## 4. Conclusions

Results indicate that under the experimental conditions employed in this study, exposure to ethanol during restricted periods of brain development is not as detrimental for DG LTP later in adulthood as a more prolonged ethanol exposure through multiple trimester equivalents of brain development. Indeed, and as one might expect, when ethanol exposure occurs only during one of the trimester equivalents, less robust effects on LTP are observed. Additionally, these results extend previous findings of sex differences with PNEE and indicate that the capacity to elicit DG LTP is differentially affected by ethanol in the male and female hippocampus.
